# Clinical Laboratory Values as Early Indicators of Ebola Virus Infection in Nonhuman Primates

**DOI:** 10.3201/eid2308.170029

**Published:** 2017-08

**Authors:** Ronald B. Reisler, Chenggang Yu, Michael J. Donofrio, Travis K. Warren, Jay B. Wells, Kelly S. Stuthman, Nicole L. Garza, Sean A. Vantongeren, Ginger C. Donnelly, Christopher D. Kane, Mark G. Kortepeter, Sina Bavari, Anthony P. Cardile

**Affiliations:** United States Army Medical Research Institute of Infectious Diseases, Fort Detrick, Frederick, Maryland, USA

**Keywords:** Ebola virus, Makona strain, Kikwit strain, Ebola virus disease, nonhuman primate models, Macaca mulatta, Macaca fascicularis, rhesus macaques, cynomolgus macaques, monkeys, early indicators of infection, survival, viruses, zoonoses, Maryland, USA, EBOV, EVD

## Abstract

The Ebola virus (EBOV) outbreak in West Africa during 2013–2016 demonstrated the need to improve Ebola virus disease (EVD) diagnostics and standards of care. This retrospective study compared laboratory values and clinical features of 3 nonhuman primate models of lethal EVD to assess associations with improved survival time. In addition, the study identified laboratory values useful as predictors of survival, surrogates for EBOV viral loads, and triggers for initiation of therapeutic interventions in these nonhuman primate models. Furthermore, the data support that, in nonhuman primates, the Makona strain of EBOV may be less virulent than the Kikwit strain of EBOV. The applicability of these findings as potential diagnostic and management tools for EVD in humans warrants further investigation.

The Ebola virus (EBOV) outbreak in West Africa during 2013–2016 highlighted the need to improve Ebola virus disease (EVD) diagnostics and standards of care (*1*). With regard to standards of care and EVD outcomes, it is important to explore potential factors associated with improved survival. In previous epidemics, dating back to 1976, EVD case fatality rates ranged from 47% to 90% (*2*). The Centers for Disease Control and Prevention (CDC) reported a crude death rate of 40% (11,310 deaths/28,616 cases) when including suspected, probable, and confirmed cases from the West Africa outbreak; however, when only confirmed cases were included, the EVD case fatality rate was 74% (11,310 deaths/15,227 cases), consistent with historical rates (*1*).

Analysis of the most recent EBOV epidemic and previous outbreaks identified predictors associated with decreased survival: high quantitative viral load (*3*–*7*); low PCR cycle threshold (*8*–*12*); age (very young and very old) (*5*,*6*,*10*,*13*–*16*); male sex (*12*,*17*); country of residence (*1*,*17*); levels of D-dimer (*18*), aspartate aminotransferase (AST) (*5*,*10*,*11*), blood urea nitrogen (BUN) (*5*), and serum creatinine (*5*,*10*); and clinical symptoms of diarrhea, pain, myalgia, hemorrhage, and difficulty breathing (*10*,*12*,*15*,*19*,*20*). Of the various EVD animal models that have been developed, those using nonhuman primates (NHPs) appear to most closely reproduce the known features of lethal disease in humans. Herein, we summarize and compare the clinical features and laboratory values of 3 NHP lethal models of EVD and explore features associated with early manifestations of infection and improved survival. Similar to Janvier et al. (*11*), who recommended the use of high AST levels in humans as a surrogate marker of EBOV viral load and, therefore, disease detection and survival, we explored whether NHP laboratory data would lend support to the use of clinical laboratory values as predictors of survival and surrogates for EBOV viral loads. Further analysis was conducted to determine if clinical laboratory values could be used for indication of infection with the goal of developing a standardized trigger for the initiation of treatment in the NHP model.

## Materials and Methods

### Animal Use and Viral Challenge

The 30 NHPs described herein served as control animals in larger therapeutic studies conducted in 2014 and 2015. We retrospectively analyzed existing data from those studies. The NHP experiments and tests for this study were performed by the same researchers at the United States Army Medical Research Institute of Infectious Diseases (USAMRIID; Fort Detrick, Frederick, MD, USA), using the same institutional standard operating procedures and processes, the same laboratory instruments, and the same standardized EBOV challenge (1,000 PFU administered intramuscularly [IM]).

We collected blood samples from all 30 NHPs immediately before virus challenge (day 0) and at 3, 5, and 7 days postinoculation (dpi). Methods for EBOV challenge of the NHPs have been described in detail (*21*). In brief, we first IM inoculated 18 rhesus macaques (*Macaca mulatta*; 3 groups of 6 animals, male and female, weighing 3.71–7.26 kg) with a target titer of 1,000 PFU of the Kikwit strain of EBOV (EBOV Kikwit; back titration titer range 950–1,358 PFU). Next, we IM inoculated 6 cynomolgus macaques (*M. fascicularis*; male and female, weighing 3.49–7.33 kg) with a target titer of 1,000 PFU of EBOV Kikwit (back titration titer 1,600 PFU). The EBOV Kikwit strain we used is a USAMRIID stock virus, EBOV H.sapiens-tc/COD/1995/Kikwit-9510621; this virus was primarily the 7U (7 uridylyls) variant at the mRNA editing site. This challenge virus was propagated from a clinical specimen by using cultured cells for a total of 4 passages. Last, we IM inoculated 6 rhesus macaques (male and female, weighing 4.79–5.40 kg) with a target titer of 1,000 PFU (back titration titer 800 PFU) of the USAMRIID stock virus EBOV H.sapiens-tc/LBR/2014/Makona (EBOV Makona); this virus was also primarily the 7U variant at the mRNA editing site. This challenge virus was propagated from a clinical specimen by using cultured cells for a total of 2 passages.

We conducted all studies in Biosafety Level 4 containment. Beginning on day 0 and continuing for the duration of the in-life phase, we recorded clinical observations and closely monitored animals at least 3 times daily for disease progression (*22*). According to protocol, we provided the NHPs with basic support with regard to pain, oral hydration, and antimicrobial drugs. We administered antimicrobial drugs only if the facility veterinarian diagnosed a secondary bacterial infection. Moribund animals were euthanized on the basis of prespecified criteria (*22*).

### Clinical Laboratory Samples

When possible, we processed and analyzed samples obtained for analyses within 6 h of collection. We used a Vitros 350 Chemistry System (Ortho Clinical Diagnostics, Raritan, NJ, USA) to analyze serum chemistries; an Advia 120 Hematology Analyzer (Siemens, Tarrytown, NY, USA) with multispecies software to analyze hematology parameters; and a Sysmex CA-1500 (Siemens) for coagulation analyses. The samples yielded a panel of 46 routine clinical laboratory values (*21*).

### Viral RNA

We used quantitative real-time PCR (qRT-PCR) to determine viral RNA copy numbers in plasma samples collected at prespecified time points (*21*). No definition has been established for high viral load in this qRT-PCR assay or in NHP models of EVD. Thus, we used a value of 9 log_10_ RNA copies/mL as a cutoff value for high versus low viral load. The rationale for this cutoff was that the median viremia value for the EBOV Kikwit–infected macaques at 5 dpi and that for the EBOV Makona–infected macaques at 7 dpi was ≈9 log_10_ copies/mL.

### Statistical Analysis

We performed univariate and multivariate regression modeling of available demographic and laboratory data and used Mann-Whitney U test and Fischer exact test, where appropriate, for the descriptive analyses. To assess the effect of variables on survival time (measured in hours), we used linear and logistic regression models with Microsoft Excel (Microsoft Corp, Redmond, WA, USA) and Stata 12 (StataCorp LLC, College Station, TX, USA). We used p value thresholds of <0.05 for statistical tests and included an adjusted p value of ≤0.001, based on a simplified Bonferroni correction, for multiple comparisons (Technical Appendix Table 1).

**Table 1:** Baseline characteristics and clinical data for 3 nonhuman primate models of lethal Ebola virus disease*

Variable	Models of infection				
	Rhesus macaque with		p value†	Cynomolgus macaque with Kikwit strain, n = 6	p value‡
Kikwit strain, n = 18	Makona strain, n = 6
Baseline characteristic					
Weight, kg	4.92	4.79	0.894	4.44	0.526
Age, y	3.94	3.49	0.575	4.92	0.107
Postchallenge clinical data					
Survival time, h	186.9	337.5	**0.005**	175.2	0.739
Clinical responsiveness score, d§					
3	0	0	None	0	None
5	0.56	0	0.078	0.55	0.729
7	1.64	0.17	**0.004**	2.60	0.059
Presence of petechial rash, d	5.65	8.17	**<0.001**	5.17	0.265
Decreased food consumption, d	5.11	8.33	**<0.001**	4.67	0.178
Presence of anuria, d	6.43	8.20	**0.008**	6.40	0.500

*Data are means. †For rhesus macaque model with Ebola virus (EBOV) Kikwit strain vs. Makona strain. Bold indicates p<0.05. ‡For rhesus macaque model with EBOV Kikwit strain vs. cynomolgus macaque model with EBOV Kikwit strain. §Clinical Responsiveness Score: 0 = active, 1 = decreased activity; 2 = mildly unresponsive (becomes active when approached), occasional prostration; 3 = moderate unresponsiveness (may require prodding to respond), weakness; 4 = moderate to severe unresponsiveness (requires prodding), moderate prostration; 5 = moribund, severe unresponsiveness, pronounced prostration.

We used the receiver operating characteristic (ROC) curve to illustrate a predictor’s performance in 2 metrics, with 1 as a tradeoff of another (e.g., sensitivity and specificity); we obtained the ROC curve by varying the laboratory threshold values of a tested predictor. Using ROC area under the curve (AUC), we evaluated the ability of routine laboratory values (alone or in combination) and log_10_ plasma viral load RNA concentrations obtained at 3, 5, or 7 dpi to predict infection. The combinations of routine laboratory values analyzed were chosen on the basis of the characteristics of individual laboratory values, clinical relevance, and likely access to the test in a field Ebola treatment unit. Day 0 laboratory values were used as baseline values. Because blood samples for viral RNA assessment and routine laboratory analysis were obtained before virus challenge, day 0 values were used to represent uninfected animals. Values obtained at 3, 5, and 7 dpi were used to represent infected animals because all NHPs were experimentally IM inoculated with EBOV (1,000 PFU target dose), and plasma viral RNA was detected in a sample from at least 1 sampling event for all NHPs included in this analysis. As part of this analysis, we combined the following variables: AST; lactate dehydrogenase (LDH); C-reactive protein (CRP); and the combination of AST, LDH, CRP, and hemoglobin (Hgb). A combined variable *C* was defined as the mean value of all normalized variables and *v_i_*:

.

*v_i_* was normalized by dividing the sum of its mean and standard deviation of baseline values (i.e., values on day 0). *s_i_* is 1 (or −1) for variables whose value increases (or decreases) after infection. *m* is the number of independent variables to be combined. Values of variable *C* were calculated for each sample on each day (including day 0) after *μ_i_*, *σ_i_*, and *s_i_* were determined. We denote the 2 combined variables as (AST+LDH+CRP) and (AST+LDH+CRP−Hgb) because AST, LDH, and CRP values increase after infection, whereas Hgb decreases after infection.

### Ethics Statement

Animal research at USAMRIID was conducted under an Institutional Animal Care and Use Committee–approved protocol in compliance with the Animal Welfare Act, US Public Health Service policy, and other federal statutes and regulations relating to animals and experiments involving animals. The facility where this research was conducted is accredited by the Association for Assessment and Accreditation of Laboratory Animal Care, International and adheres to principles stated in the Guide for the Care and Use of Laboratory Animals, National Research Council, 2011.

## Results

**Figure 1:**
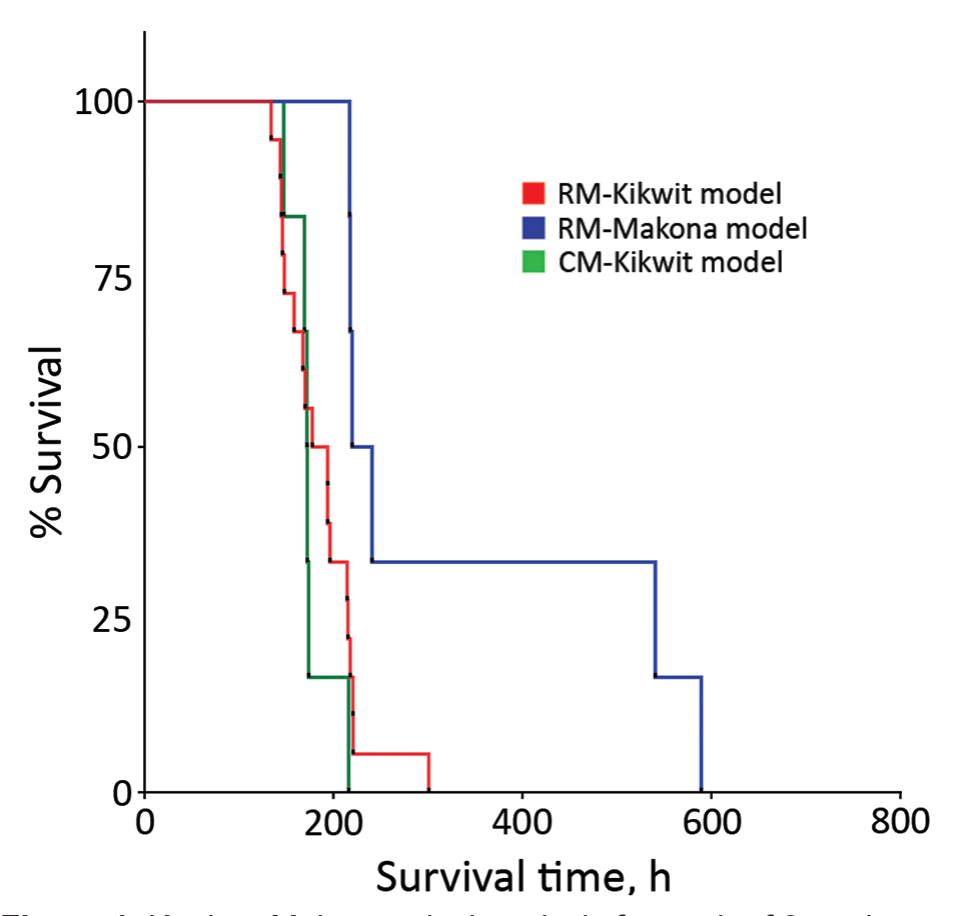
Kaplan–Meier survival analysis for each of 3 nonhuman primate models of Ebola virus disease: rhesus macaque model with EBOV Kikwit strain (n = 18 monkeys); rhesus macaque model with EBOV Makona strain (n = 6 monkeys); and cynomolgus macaque model with EBOV Kikwit strain (n = 6 monkeys). Overall comparison of the 3 Kaplan–Meier survival curves yielded a statistically significant value (p = 0.007) using the Mantel–Cox log-rank test. CN-Kikwit, cynomolgus macaque model of EBOV Kikwit strain; EBOV, Ebola virus; RM-Kikwit, rhesus macaque model of EBOV Kikwit strain; RM-Makona, rhesus macaque model of EBOV Makona strain.

**Figure 2:**
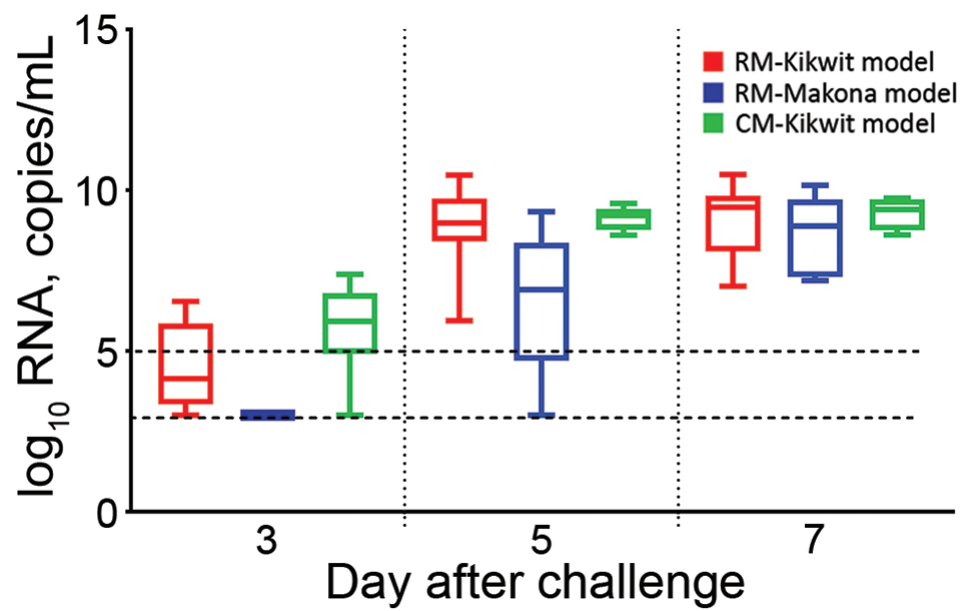
log_10_ RNA level, by day after EBOV challenge, for each of 3 nonhuman primate models of Ebola virus disease. Box and whisker plots were created by using the available data for each day. Boxes indicate range from 25th (bottom line) to 75th (top line) percentiles; horizontal line within each box indicates median; whiskers indicate entire range of values (maximum to minimum). Dashed lines indicate limit of detection (LOD) (bottom line, 3.0 log_10_ RNA copies/mL) and lower limit of quantification (LLOQ) (top line, 5.0 log_10_ RNA copies/mL) for the assay. Values below the LOD were assigned the value 3.0 log_10_ RNA copies/mL; values between the LLOQ and the LOD were assigned the actual measured value. CM-Kikwit, cynomolgus macaque model of EBOV Kikwit strain; EBOV, Ebola virus; RM-Kikwit, rhesus macaque model of EBOV Kikwit strain; RM-Makona, rhesus macaque model of EBOV Makona strain.

Similarities and differences in baseline summary characteristics of the 3 groups of NHPs are worth noting (Tables 1, 2) as well as similarities and differences in Kaplan–Meier survival analysis (Figure 1). Median RNA viral loads peaked in all 3 NHP groups at 7 dpi (Figure 2), but RNA viral loads for some EBOV Kikwit–infected NHPs peaked at 5 dpi. At each time point, viremia values ranged widely between the 3 groups. The only significant differences between the NHP groups were at 3 dpi, when the EBOV Kikwit–infected rhesus macaques had higher mean log_10_ RNA values (4.50 RNA copies/mL [range <3.0 to 6.54]) than the EBOV Makona–infected rhesus macaques, all of whom had log_10_ RNA values below the limit of detection (<3.00 RNA copies/mL; p<0.001), and at 5 dpi, when the EBOV Kikwit–infected rhesus macaques had higher mean log_10_ RNA values (8.94 RNA copies/mL [range 5.94 to 10.47]) than the EBOV Makona–infected rhesus macaques (6.57 RNA copies/mL [range <3.0 to 9.33]; p<0.049).

**Table 2:** Results of selected laboratory tests for 3 nonhuman primate models at various days after challenge with EBOV*

Laboratory variable, d	Models of infection				
	Rhesus macaque with		p value‡	Cynomolgus macaque with Kikwit strain, mean (range), n = 6	p value§
	Kikwit strain, mean (range), n = 18†	Makona strain, mean (range), n = 6			
BUN, mg/dL					
0	16.1 (11–22)	15.2 (10–19)	0.544	17.8 (16–23)	0.217
3	15.3 (11–20)	14.3 (8–19)	0.615	17.3 (13–21)	0.215
5	20.0 (10–39)	14.2 (10–17)	0.365	38.5 (15–116)	0.124
7	58.7 (11–108)	17.2 (11–24)	**0.050**	112.6 (58–135)	**0.015**
Creatinine, mg/dL					
0	0.6 (0.5–0.8)	0.5 (0.5–0.6)	**0.012**	0.6 (0.5–0.9)	0.871
3	0.6 (0.5, 0.8)	0.5 (0.4–0.6)	**0.030**	0.6 (0.4–0.9)	0.662
5	1.1 (0.6–2.6)	0.6 (0.5–0.7)	**0.005**	1.8 (0.8–5.2)	0.094
7	2.3 (0.7–5.6)	0.8 (0.7–1.0)	0.055	24.3 (1.7 to >56.0)	**0.037**
AST, U/L					
0	37.9 (22– 62)	35.8 (26– 53)	0.702	64.7 (37– 151)	0.192
3	49.4 (32, 74)	42.0 (31–57)	0.230	95.7 (47–145)	**0.002**
5	411.6 (46–1,716)	47.0 (33–56)	**0.001¶**	423.2 (116–743)	0.386
7	991.4 (145–1,585)	244.5 (113–398)	**0.009**	1,626.6 (752 to >3,400)	0.624
ALT, U/L					
0	32.1 (10–64)	17.5 (7–27)	**0.009**	53.8 (36–94)	0.078
3	45.2 (10–87)	25.0 (10–38)	**0.009**	60.2 (51–81)	**0.028**
5	137.2 (19–554)	29.0 (13–46)	**0.008**	87.8 (51–138)	0.790
7	299.4 (68–606)	67.2 (21–108)	**0.016**	610.0 (154–2,087)	0.955
CRP, mg/L					
0	5.6 (0–20)	5.2 (5–6)	0.651	6.8 (4–11)	0.385
3	10.1 (5–31)	5.2 (5–6)	**0.013**	19.7 (8–59)	**0.047**
5	71.2 (43–83)	17.2 (6–44)	**<0.001¶**	73.8 (70–78)	0.764
7	59.5 (44–74)	57.3 (32–71)	0.960	48.2 (13–72)	0.533
LDH, IU/L					
0	510.8 (366–679)	456.0 (390–537)	0.083	964.7 (653–1,267)	**0.008**
3	641.0 (381–829)	563.3 (454–775)	0.110	1,511.5 (894–2,532)	**<0.001¶**
5	3,897.2 (670 to >9,000)	700.8 (551–826)	**0.001¶**	5,799.8 (1,667 to >9,000)	0.229
7	7,965.7 (1,531 to >9,000)	5,524.3 (1,562 to >9,000)	**0.042**	9,000 (>9,000 to >9,000)	0.353
CPK, U/L					
0#	435.2 (55–915)	214.7 (84–395)	**0.006**	ND	
3	507.3 (181–874)	557.0 (333–897)	0.594	ND	
5	1,721.3 (183–5157)	494.5 (287–755)	**0.014**	ND	
7	4,599.1(320 to >6,400)	2,459.3 (700–5,692)	0.065	ND	
Platelets, × 10^3^/mm^3^					
0	347.5 (240–502)	274.3 (220–318)	**0.002**	312.5 (278–373)	0.102
3	330.4 (223–557)	285.7 (244–330)	0.193	288.2 (237–352)	0.217
5	172.0 (91–303)	253.7 (199–286)	**0.006**	197.3 (144–312)	0.350
7	89.6 (34–161)	112.3 (26–191)	0.482	142.4 (106–195)	**0.047**
PT, s					
0	11.2 (10.4–14.9)	11.4 (10.8–12.2)	0.374	10.6 (9.8–11.3)	**0.010**
3	10.7 (9.8–12.7)	10.9 (10.1–12.0)	0.365	10.4 (9.7–10.9)	0.545
5	13.9 (10.9–18.1)	10.4 (10.0–10.9)	**<0.001¶**	14.1 (12.6–17.1)	0.739
7	15.7 (12–19.6)	12.4 (11.7–13.8)	**0.004**	18.0 (14.6–22.8)	0.282
APTT, s					
0	27.0 (24.5–32.0)	27.5 (26.8–29.6)	0.440	25.8 (24.4–27.5)	0.095
3	26.7 (23.8–31.5)	26.1 (25.0–28.5)	0.841	27.1 (25.0–32.9)	0.947
5	43.4 (31.5–62.6)	27.6 (24.7–31.8)	**<0.001¶**	41.2 (35.2–48.4)	0.571
7	60.4 (42.3–111.1)	41.9 (34.9–47.6)	**0.012**	62.5 (51.3–67.4)	0.532
AT, %					
0	101.8 (85.8–116.9)	105.7 (90.5–121.6)	0.450	100.5 (92.0–118.6)	0.768
3	104.2 (76.0–127.3)	110.0 (98.8–119.8)	0.286	103.0 (92.8–115.3)	0.689
5	76.9 (55.5–100.9)	113.8 (103.1–129.2)	**<0.001¶**	73.5 (70.4–83.3)	0.505
7	67.7 (38.5–94.8)	103.1 (95.5–116.0)	**<0.001¶**	49.4 (34.0–55.9)	**0.031**

*ALT, alanine aminotransferase; APTT, activated partial thromboplastin time; AST, aspartate aminotransferase; AT, antithrombin; BUN, blood urea nitrogen; CPK, creatine phosphokinase; CRP, C-reactive protein; EBOV, Ebola virus; LDH, lactate dehydrogenase; ND, not done; PT, prothrombin time. †Results for 6 macaques in the EBOV Kikwit strain group were previously reported as a mean difference from day 7 to day 0 (*28*). ‡For results for rhesus macaque model of infection with EBOV Kikwit strain vs. Makona strain. Bold indicates p<0.05. §For rhesus macaque model with EBOV Kikwit strain vs. cynomolgus macaque model with EBOV Kikwit strain. Bold indicates p<0.05. ¶Adjusted p value of <0.001, based upon a simplified Bonferroni correction for multiple comparisons. #In the 24 nonhuman primates infected with EBOV for whom CPK values were analyzed, 16 (67%) had levels >5,000 U/L during the course of disease.

**Figure 3:**
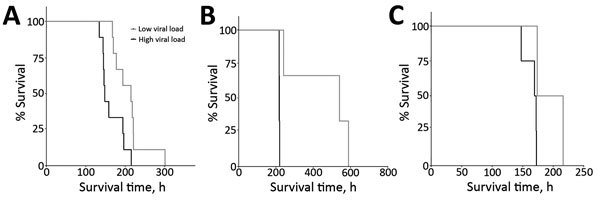
Survival curves, stratified by high (>9 log_10_ RNA copies/mL) and low (<9 log_10_ RNA copies/mL) viral loads, for each of 3 nonhuman primate models of Ebola virus disease. A) Comparison of survival on postinoculation day 5 for rhesus macaques infected with the Kikwit strain of Ebola virus (EBOV). Median survival time was 148.0 hours for macaques with high viral loads (n = 9) and 214.6 hours for macaques with low viral loads (n = 9). Comparison of the 2 survival curves yielded a statistically significant value (p = 0.010 by Mantel-Cox log-rank test). B) Comparison of survival on postinoculation day 7 for rhesus macaques infected with the Makona strain of EBOV. Median survival time was 217.7 hours for macaques with high viral loads (n = 3) and 540.4 hours for macaques with low viral loads (n = 3). Comparison of the 2 survival curves yielded a statistically significant value (p = 0.025 by Mantel-Cox log-rank test). C) Comparison of survival on postinoculation day 5 for cynomolgus macaques infected with the Kikwit strain of EBOV. Median survival time was 170.6 hours for macaques with high viral loads (n = 4) and 195.0 hours for macaques with low viral loads (n = 2). Comparison of the 2 survival curves yielded a nearly statistically significant value (p = 0.074 by Gehan–Breslow–Wilcoxon test).

In the EBOV Kikwit–infected rhesus macaques, the median survival time was significantly different between animals with high viral loads (214.6 h) and those with low viral loads (148.0 h) (p = 0.013) (Figure 3). In general, viral load correlated with survival time as early as 3 dpi for EBOV Kikwit–infected rhesus macaques (r = 0.57; p = 0.013); 5 dpi for EBOV Kikwit–infected cynomolgus macaques (r = 0.75; p = 0.084); and 7 dpi for EBOV Makona–infected rhesus macaques (r = 0.90; p = 0.016).

### EBOV Kikwit–Infected Rhesus Macaques versus EBOV Makona–Infected Rhesus Macaques

Among rhesus macaques, survival time was longer for those infected with EBOV Makona (337.5 hours) than those infected with EBOV Kikwit (186.9 hours; p = 0.005) (Table 1). Clinical assessments showed significant differences between clinical disease progression in EBOV Kikwit–infected and EBOV Makona–infected NHPs at 5 and 7 dpi (Table 1). In addition, we found significant differences in laboratory assessments at 5 and 7 dpi (Table 2). And, at 5 dpi, log_10_ plasma concentrations of viral RNA were significantly higher among EBOV Kikwit–infected rhesus macaques (8.94 RNA copies/mL) than among EBOV Makona–infected rhesus macaques (6.57 RNA copies/mL; p = 0.049).

### EBOV Kikwit–Infected Rhesus Macaques versus EBOV Kikwit–Infected Cynomolgus Macaques

We found no significant difference in survival time between EBOV Kikwit–infected rhesus (186.9 hours) and cynomolgus (175.2 hours) macaques. In addition, at 5 and 7 dpi, we found no significant difference in clinical findings between these 2 groups (Table 1). However, there were subtle differences in laboratory values (Table 2). Compared with rhesus macaques, cynomolgus macaques had worsened markers of renal function at 7 dpi, as evidenced by mean laboratory values: BUN levels of 112.6 mg/dL for cynomolgus macaques versus 58.7 mg/dL for rhesus macaques (p = 0.015); serum creatinine levels of 24.3 mg/dL for cynomolgus macaques versus 2.3 mg/dL for rhesus macaques (p = 0.037); and serum potassium levels of 6.2 mEq/L for cynomolgus macaques versus 4.2 mEq/L for rhesus macaques. Furthermore, at 7 dpi, mean platelet counts tended to be lower for rhesus (89.6 × 10^3^/mm^3^) than cynomolgus (142.4 × 10^3^/mm^3^; p = 0.047) macaques (Table 2).

### Regression Analyses for Predicting Viral Load from Routine Laboratory Values

Due to small sample sizes, we limited our presentation of regression analyses to the 18 EBOV Kikwit–infected rhesus macaques. In a univariate regression model, log_10_ plasma concentrations of viral RNA correlated significantly with survival time at peak viremia (5–7 dpi) and at 3, 5, and 7 dpi (Technical Appendix). At 5 dpi, the following laboratory values correlated significantly with time to death and with plasma viral load: platelet counts; prothrombin time; and levels of AST; alanine aminotransferase (ALT); LDH; and creatine phosphokinase (CPK) (Technical Appendix). Similarly, LDH and CPK values at 7 dpi correlated significantly with time to death and with log_10_ viral RNA (Technical Appendix).

### ROC Curve Analyses for Assessing Clinical Laboratory Values as Early Indicators of EBOV Infection

In the following datasets, ROC curve analysis yielded the best available laboratory predictors as signs of EBOV infection at 3, 5, and 7 dpi: log_10_ RNA, AST, ALT, CRP, LDH, CPK, and Hgb (Technical Appendix Table 2). log_10_ concentrations of viral RNA outperformed all other individual laboratory values as a predictor of EBOV infection. However, ROC AUC values for log_10_ RNA were only slightly better than those for LDH, CRP, and AST (Technical Appendix Table 2). In fact, when the 3 chemistries were combined (AST+LDH+CRP), they performed almost as well as log_10_ RNA values in all 3 NHP models. When we compared the combined predictor AST+LDH+CRP−Hgb with log_10_ RNA values at 3 dpi, it outperformed log_10_ RNA in all 3 NHP models. For example, at 3 dpi in the EBOV Kikwit–infected rhesus macaque model, ROC AUC was 0.83 for log_10_ RNA and 0.93 for the combined predictor AST+LDH+CRP−Hgb.

## Discussion

Unlike some other reports showing abnormal laboratory values in NHP EVD models, we have presented our findings in a systematic format concentrating on laboratory values that we think reflect EVD pathogenesis, are easily translatable to human disease, and are potentially available in the human clinical setting. A better understanding of EBOV NHP models will enhance characterization of the disease and facilitate standardization of the models to support possible future vaccine and therapeutic drug submissions under the Food and Drug Administration Animal Rule (https://www.fda.gov/downloads/Drugs/GuidanceComplianceRegulatoryInformation/Guidances/UCM399217.pdf). Similar to what has been reported in human EVD (*3*–*12*), our findings demonstrate that a lower plasma concentration of viral RNA predicted increased survival time in the 3 NHP models we assessed.

Marzi et al. (*23*) observed that disease progression in EBOV Makona–infected cynomolgus macaques was delayed compared with that in cynomolgus macaques infected with the EBOV Mayinga strain. Wong et al. (*24*) compared infections with EBOV Kikwit with infections with 2 different EBOV Makona strains in rhesus macaques and found that the EBOV Makona strains were either similar in virulence or more virulent than the EBOV Kikwit strain they were using. We observed that disease progression in EBOV Makona–infected rhesus macaques was delayed compared with that in EBOV Kikwit–infected rhesus macaques; the observation was supported by viral load data, clinical assessments, and laboratory values. This finding might suggest that, in NHPs, the EBOV Makona strain we used is somewhat less virulent than the EBOV Kikwit strain we used; although, in rhesus macaques, 1,000 PFU of EBOV Makona still resulted in death among all untreated animals. Data are insufficient to determine the relative pathogenicity of EBOV Makona in comparison with that of other EBOV strains. To make this determination, further studies are needed, taking into consideration different EBOV strains and quasispecies.

The difference in survival time between EBOV Kikwit–infected rhesus and cynomolgus macaques (11.7 hours) was not significant; rhesus macaques survived longer. Of interest, at 7 dpi, cynomolgus macaques demonstrated increased impairment of renal function (as determined by BUN and creatinine levels) compared with that for rhesus macaques. This finding does not appear to be associated with a significant observable difference in oral fluid consumption between the animals, and the pathogenesis merits further study.

The search for reliable biomarkers for early diagnosis of EBOV infection and predictors of survival has been a high priority (*25*,*26*), but reverse transcription PCR (RT-PCR) remains the reference standard for EVD diagnosis (*27*). ROC analysis demonstrated that, in all 3 NHP models, qRT-PCR outperformed all individual laboratory values with regard to EVD confirmation. It is noteworthy that the combination of AST+LDH+CRP−Hgb values outperformed qRT-PCR as a laboratory sign of Ebola virus infection at 3 dpi in all 3 NHP models. This could be an important finding and potentially serve as a trigger to treat NHPs in therapeutic studies of IM administered EBOV Kikwit.

Currently, there are no standardized triggers for the initiation of treatment of EBOV-infected NHPs in therapeutics studies. Various time points after virus exposure have been used for therapeutics initiation in NHP models of EBOV infection (*28*–*30*). One study used a positive RT-PCR result plus documented fever of >1.5°C above baseline for 1 hour as a prespecified trigger to treat (*31*). However, it is logistically difficult to obtain timely PCR results in a Biosafety Level 4 laboratory (especially for a large study), and implantation of a telemetry device would be required for optimal fever detection. Thus, standard clinical laboratory values may be a more practical trigger to treat. For example, a calculator (spreadsheet or smartphone application) could be developed to calculate a combined variable value (e.g., AST+LDH+CRP−Hgb) from clinical laboratory values. This approach would be similar to a disease severity smartphone application advocated by Colubri et al. (*32*) for use with human EBOV patients. Once the threshold laboratory value is reached or exceeded, the therapeutic could be initiated. We intend to conduct a follow-up ROC AUC analysis with a larger sample size to further validate and optimize these preliminary findings.

Our finding that, in lieu of viral load, laboratory values at 5 dpi could potentially predict survival duration is not entirely surprising given that Warren et al. (*28*) published NHP data that showed the course of EBOV viral load is mirrored by the course of clinical chemistries in the setting of successful EVD treatment using the nucleotide prodrug GS-5734. Although it has been shown that AST levels can predict survival in EBOV-infected humans (*5*,*10*,*11*), we found that LDH may be a better predictor of survival time in NHP models using IM administered EBOV. In all 3 models in our study, LDH and viral load significantly increased at 5 dpi in EBOV Kikwit–infected rhesus and cynomolgus macaques and at 7 dpi in EBOV Makona–infected rhesus macaques. In the EBOV Kikwit–infected rhesus macaque model, LDH values at 5 dpi correlated with viral load and survival time at 5 dpi. Incidentally, LDH has been shown to correlate with survival time in humans with Crimean-Congo hemorrhagic fever, severe fever with thrombocytopenia syndrome, and Dengue virus infection (*33*–*36*).

LDH is abundant in the cytoplasm of all human cells and helps catalyze the conversion of pyruvate to lactate, the last step of glycolysis (*37*). Markedly elevated levels of LDH are often seen in association with cardiogenic shock; hepatic ischemia or necrosis; and intestinal and/or mesenteric ischemia or necrosis (*36*,*38*). However, no signs of cardiogenic shock, severe chemical transaminitis corresponding to hepatic ischemia or necrosis, or severe hemolysis were observed at 5 dpi in EBOV Kikwit–infected NHPs or at 7 dpi in EBOV Makona–infected NHPs. One possibility is that the NHPs were experiencing either intestinal and/or mesenteric ischemia or necrosis, conditions that have been seen in other viral infections (*39*,*40*) and which may, as postulated by Lynn (*41*), precipitate late Ebola sepsis-like syndrome.

Kortepeter et al. (*42*) reported that, in a rhesus macaque model, animals lethally challenged with EBOV Kikwit experienced a rapid increase in plasma viral RNA beginning at 4 dpi and a rapid increase in serum lactate beginning at 7 dpi. In humans, serum lactate levels have been shown to correlate with serum LDH levels (*43*), and both have independently been associated with death (*44*–*47*). Thus, further study is needed of lactate and LDH levels in humans and NHPs with EVD.

A key caveat to our analysis is that our data reflect a retrospective analysis of NHPs used as controls in 5 different studies of IM administered EBOV. The trajectory of early increases in clinical laboratory values, especially CPK and LDH, could be affected by the IM route of EBOV administration. However, Johnson et al. (*48*) reported similar early increases in LDH and CPK in rhesus macaques in an aerosol model of EBOV Zaire infection. In future analyses, we intend to explore how the route of EBOV administration affects changes in clinical laboratory values. Another limitation to this analysis is that survival time was determined to be time to euthanasia. The strict adherence to the USAMRIID euthanasia criteria, in the setting of 3 clinical assessments daily, supports the supposition that time to euthanasia approximates time to death. Another limitation is that, in this dataset, all animals died, so we were only able to look at survival time. Therefore, we were unable to derive odds ratios for individual variables to assess predictors of death. Future analyses of datasets that include NHPs that survived EBOV infection, with or without treatment with a therapeutic product, will be useful to identify such predictors. Although our analysis included a large number of NHPs (N = 30), we also acknowledge that this retrospective analysis was primarily hypothesis-generating and that there were no prespecified hypotheses. We used p value thresholds of <0.05 for our statistical tests and included an adjusted p value of <0.001 on the basis of a simplified Bonferroni correction for multiple comparisons.

Much can be learned from a critical analysis of EBOV NHP models. Our data support the finding that the virulence of the EBOV Makona strain used in our study may be decreased as compared to that of the EBOV Kikwit strain we used. We did not find a statistically significant difference in survival time when comparing rhesus to cynomolgus macaques in the EBOV Kikwit model, although there were subtle differences in some of the laboratory values. In addition, our data support EBOV studies in humans that indicate basic laboratory values could potentially be used as surrogate markers for viral load and, thus, disease detection and survival. However, validation of this approach in the human clinical setting would also require a comparison with clinical laboratory values associated with endemic diseases present in a given geographic area, such as malaria, rickettsial illnesses, and diseases caused by other hemorrhagic fever viruses. In addition, a combined score of AST, LDH, CRP, and Hgb values could be further evaluated as a trigger to treat NHPs in therapeutics studies of IM administered EBOV. Further work in the NHP model of EVD with regard to clinical and laboratory markers would ideally lead to improvements in predicting survival time in EBOV-infected NHPs and enhancements in the treatment of disease in NHPs, with potential applicability to the management of human EVD.

Technical Appendix Additional materials and methods and results of various analyses in a study of clinical laboratory values as early indicators of Ebola virus infection in nonhuman primates.
