# Supplementary material for: Clinical Laboratory Values as Early Indicators of Ebola Virus Infection in Nonhuman Primates
Source: Emerg Infect Dis. 2017 Aug;23(8):1316–24. doi: 10.3201/eid2308.170029 (PMC5547776; doi:10.3201/eid2308.170029)
Supplement: Technical Appendix — Additional materials and methods and results of various analyses in a study of clinical laboratory values as early indicators of Ebola virus infection in nonhuman primates. [file 17-0029-Techapp-s1.pdf]

Article DOI: https://doi.org/10.3201/eid2308.170029

Clinical Laboratory Values as Early Indicators of Ebola Virus Infection in Nonhuman Primates

Technical Appendix

Laboratory Data for Table 1 in Main Text

A Vitros 350 Chemistry System (Ortho Clinical Diagnostics, Raritan, NJ, USA) was used to analyze the following serum chemistry parameters: sodium, potassium, chloride, ALT, AST, alkaline phosphatase, LDH, γ-glutamyl transferase, direct and indirect bilirubin, amylase, lipase, calcium, phosphorus, BUN, total protein, albumin, globulin, glucose, cholesterol, triglycerides, CPK, and CRP. An Advia 120 Hematology Analyzer (Siemens, Tarrytown, NY, USA) was used with multispecies software to analyze the following hematology parameters: red blood cell count, hemoglobin, % hematocrit, mean corpuscular volume, mean corpuscular hemoglobin, mean cell hemoglobin concentration, red cell distribution width, mean platelet volume, and reticulocyte count. Erythrocytes included leukocyte count and absolute counts for neutrophils, lymphocytes, monocytes, eosinophils, and basophils. A Sysmex CA-1500 Analyzer (Siemens, Tarrytown, NY, USA) was used for coagulation analysis of the following parameters: PT, APTT, thrombin time, fibrinogen concentration, AT, and D-dimer concentration (*1*).

Reference

1. Martins K, Cooper C, Warren T, Wells J, Bell T, Raymond J, et al. Characterization of clinical and immunological parameters during Ebola virus infection of rhesus macaques. Viral Immunol. 2015;28:32–41. PubMed http://dx.doi.org/10.1089/vim.2014.0085

Technical Appendix Table 1. Regression analysis for EBOV-Kikwit (N = 18) yielding correlation to time to death and correlation to log_{10} RNA*

Variable	P-value, time to death	P-value, log_{10} RNA
Log_{10} Viral Load (RNA), Day 3	**0.013**	NA
Log_{10} Viral Load (RNA), Day 5	**<0.001†**	NA
Log_{10} Viral Load (RNA), Day 7	**0.008**	NA
Peak Log_{10} Viral Load (RNA), Days 5–7	**0.003**	NA
BUN Day 5	**0.001†**	0.227
BUN Day 7	**0.027**	0.295
Cr Day 5	**0.003**	0.105
Cr Day 7	0.145	0.245
AST Day 5	**0.005**	**0.001†**
AST Day 7	**0.007**	0.068
ALT Day 5	**0.016**	**0.001†**
ALT Day 7	0.138	0.353
CRP Day 5	0.994	0.144
CRP Day 7	0.136	0.137
LDH Day 5	**<0.001†**	**<0.001†**
LDH Day 7	**<0.001†**	**0.003**
CPK Day 5	**0.012**	**0.007**
CPK Day 7	**0.021**	**0.006**
PLT Day 5	**0.007**	**0.002**
PLT Day 7	0.169	0.966
PT Day 5	**0.014**	**0.043**
PT Day 7	**0.002**	0.189
APTT Day 5	0.118	0.078
APTT Day 7	0.126	0.652
AT % Day 5	0.117	0.098
AT % Day 7	**0.017**	0.636

*ALT, alanine aminotransferase; APTT, activated partial thromboplastin time; AST, aspartate aminotransferase; AT, antithrombin; BUN, blood urea nitrogen; CPK, Creatine Phosphokinase; Cr, creatinine; CRP, C-reactive protein; LDH, lactate dehydrogenase; PLT, platelet; PT, prothrombin time. Bold face $p < 0.05$ values for easy identification.

†Adjusted P-value of $\leq$0.001 based upon a simplified Bonferroni correction for multiple comparisons.

Technical Appendix Table 2. ROC AUC, cutoff values, sensitivity, and specificity associated with select laboratory parameters at Days 3, 5, and 7 post Ebola virus infection*

	ROC AUC [95% CI]			Laboratory cutoff value			Sensitivity			Specificity		
Parameter	Day 3	Day 5	Day 7	Day 3	Day 5	Day 7	Day 3	Day 5	Day 7	Day 3	Day 5	Day 7
All 3 Models, n = 30												
Log10 RNA	0.83 [0.83,0.84]	0.97 [0.97,0.97]	1.00 [1.00,1.00]	≥3.31	≥5.26	≥7.01	0.83	0.97	1	1	1	1
AST	0.71 [0.71,0.71]	0.91 [0.91,0.92]	1.00 [1.00,1.00]	≥30	≥103	≥113	1	0.73	1	0.23	0.97	0.97
ALT	0.67 [0.67,0.67]	0.79 [0.79,0.79]	0.93 [0.93,0.93]	≥10	≥45	≥68	1	0.73	0.86	0.03	0.8	0.93
CRP	0.73 [0.73,0.73]	0.99 [0.99,0.99]	1.00 [1.00,1.00]	≥7	≥23	≥13	0.6	0.87	1	0.87	1	0.97
LDH	0.73 [0.73,0.73]	0.94 [0.94,0.94]	1.00 [1.00,1.00]	≥518	≥670	≥1531	0.87	0.97	1	0.53	0.8	1
CPK	0.69 [0.69,0.69]	0.83 [0.83,0.84]	0.96 [0.96,0.96]	≥268	≥755	≥1080	0.92	0.63	0.88	0.46	0.96	1
Hgb	0.70 [0.70,0.70]	0.80 [0.80,0.81]	0.84 [0.84,0.84]	≤13.2	≤13.6	≤15.5	0.03	0.07	0.05	0.93	1	1
AST+LDH+CRP†	0.78 [0.78,0.78]	0.98 [0.98,0.98]	1.00 [1.00,1.00]	≥0.71	≥2.18	≥3.42	0.77	0.83	1	0.73	1	1
AST+LDH+CRP-Hgb†	0.84 [0.84,0.84]	0.98 [0.98,0.98]	1.00 [1.00,1.00]	≥-0.12	≥0.58	≥2.58	0.67	0.86	1	0.90	1	1
Kikwit Rhesus, n = 18												
Log10 RNA	0.83 [0.83,0.84]	1.00 [1.00,1.00]	1.00 [1.00,1.00]	≥3.31	≥5.94	≥7.01	0.83	1	1	1	1	1
AST	0.75 [0.74,0.75]	0.98 [0.98,0.98]	1.00 [1.00,1.00]	≥65	≥103	≥145	0.22	0.89	1	1	0.89	1
ALT	0.71 [0.71,0.72]	0.84 [0.84,0.84]	1.00 [1.00,1.00]	≥31	≥80	≥68	0.89	0.56	1	0.61	0.56	1
CRP	0.78 [0.78,0.79]	1.00 [1.00,1.00]	1.00 [1.00,1.00]	≥7	≥43	≥44	0.67	1	1	0.89	1	1
LDH	0.83 [0.83,0.83]	1.00 [1.00,1.00]	1.00 [1.00,1.00]	≥612	≥837	≥1531	0.67	0.94	1	0.89	0.94	1
CPK	0.61 [0.61,0.61]	0.86 [0.86,0.87]	0.93 [0.93,0.94]	≥181	≥781	≥2382	1	0.78	0.9	0.11	0.78	0.9
Hgb	0.71 [0.71,0.71]	0.77 [0.77,0.77]	0.93 [0.93,0.93]	≤13.2	≤13.6	≤10.2	0.06	0.11	0.36	0.89	0.11	0.36
AST+LDH+CRP†	0.87 [0.87,0.87]	1.00 [1.00,1.00]	1.00 [1.00,1.00]	≥0.85	≥2.31	≥4.00	0.89	1	1	0.78	1	1
AST+LDH+CRP-Hgb†	0.93 [0.93,0.93]	1.00 [1.00,1.00]	1.00 [1.00,1.00]	≥0.04	≥1.52	≥3.3	0.78	1	1	0.94	1	1
Makona Rhesus, n = 6‡												
Log10 RNA	0.67 [0.59,0.75]	0.83 [0.77,0.90]	1.00 [1.00,1.00]	NA	≥5.26	≥7.19	NA	0.83	1	NA	1	1
AST	0.68 [0.66,0.74]	0.79 [0.78,0.85]	1.00 [1.00,1.00]	≥31	≥33	≥113	1	1	1	0.5	0.5	1
ALT	0.75	0.78	0.94	≥28	≥31	≥32	0.5	0.67	0.83	1	1	1

	ROC AUC [95% CI]			Laboratory cutoff value			Sensitivity			Specificity		
Parameter	Day 3	Day 5	Day 7	Day 3	Day 5	Day 7	Day 3	Day 5	Day 7	Day 3	Day 5	Day 7
	[0.74,0.81]	[0.75,0.84]	[0.93,0.97]									
CRP	0.50	0.99	1.00	≥6	≥8	≥32	0.17	0.83	1	0.83	1	1
	[0.50,0.50]	[0.98,1.00]	[1.00,1.00]									
LDH	0.86	1.00	1.00	≥454	≥551	≥1562	1	1	1	0.67	1	1
	[0.84,0.92]	[1.00,1.00]	[1.00,1.00]									
CPK	0.97	0.94	1.00	≥462	≥287	≥700	0.83	1	1	1	0.83	1
	[0.96,0.99]	[0.93,0.97]	[1.00,1.00]									
Hgb	0.74	0.79	0.85	≤11	≤10.8	≤10.7	0.83	0.67	0.5	0.17	0.17	0.17
	[0.71,0.80]	[0.78,0.84]	[0.82,0.90]									
AST+LDH+CRP†	0.75	1.00	1.00	≥0.82	≥1.06	≥5.34	1	1	1	0.5	1	1
	[0.72,0.80]	[1.00,1.00]	[1.00,1.00]									
AST+LDH+CRP-Hgb†	0.78	1.00	1.00	≥-0.03	≥0.2	≥4.49	1	1	1	0.67	1	1
	[0.77,0.83]	[1.00,1.00]	[1.00,1.00]									
Kikwit Cynomolgus, n = 6§												
Log10 RNA	0.83	1.00	1.00	≥5.6	≥8.6	≥8.61	0.83	1	1	1	1	1
	[0.77,0.90]	[1.00,1.00]	[1.00,1.00]									
AST	0.75	0.97	1.00	≥85	≥278	≥752	0.83	0.83	1	0.83	1	1
	[0.68,0.80]	[0.96,0.99]	[1.00,1.00]									
ALT	0.69	0.83	1.00	≥51	≥51	≥154	1	1	1	0.67	0.67	1
	[0.64,0.78]	[0.81,0.89]	[1.00,1.00]									
CRP	0.90	1.00	1.00	≥8	≥70	≥13	1	1	1	0.67	1	1
	[0.88,0.94]	[1.00,1.00]	[1.00,1.00]									
LDH	0.81	1.00	1.00	≥1431	≥1667	≥9000	0.5	1	1	1	1	1
	[0.73,0.86]	[1.00,1.00]	[1.00,1.00]									
Hgb	0.72	0.94	0.60	≤11.6	≤11.9	≤13.1	0.83	0.17	0.4	0.33	0.33	1
	[0.68,0.79]	[0.93,0.97]	[0.61,0.73]									
AST+LDH+CRP†	0.92	1.00	1.00	≥1.32	≥3.49	≥7.4	0.67	1	1	1	1	1
	[0.87,0.95]	[1.00,1.00]	[1.00,1.00]									
AST+LDH+CRP-Hgb†	0.94	1.00	1.00	≥0.15	≥2.67	≥6.55	0.83	1	1	1	1	1
	[0.91,0.96]	[1.00,1.00]	[1.00,1.00]									

*All p-values for each ROC AUC (XXX) value were <0.001. XXX. Log10 RNA ribonucleic acid, *ALT, alanine aminotransferase; AST, aspartate aminotransferase; CPK, Creatine Phosphokinase; Cr, creatinine; CRP, C-reactive protein; Hgb hemoglobin, LDH, lactate dehydrogenase;
†Laboratory cutoff value calculated by maximizing the sum of sensitivity and specificity.
‡For Makona infected Rhesus viremia was not detected on day 3 post-infection.
§For Kikwit–infected cynomolgus macaques CPK creatinine phosphokinase was not collected.
